# Association Between Diabetes Mellitus and Carpal Tunnel Syndrome: Results From the United States National Ambulatory Medical Care Survey

**DOI:** 10.7759/cureus.13844

**Published:** 2021-03-12

**Authors:** Jason Low, Adrian Kong, Grettel Castro, Pura Rodriguez de la Vega, Juan Lozano, Marcia Varella

**Affiliations:** 1 Department of Translational Medicine, Florida International University Herbert Wertheim College of Medicine, Miami, USA; 2 Department of Orthopedics, Florida International University Herbert Wertheim College of Medicine, Miami, USA

**Keywords:** carpal tunnel syndrome, diabetes mellitus, association, national ambulatory medical care survey

## Abstract

Background

Carpal tunnel syndrome (CTS) is the most common compression neuropathy in the upper limb. While various risk factors have been linked to CTS, the role of diabetes mellitus (DM) in the development of CTS remains unclear. Previous studies have failed to consistently demonstrate a clear association between DM and CTS due to variations based on the geographic setting and differences in the study design. The objective of this study was to assess if there is an association between DM and CTS using population-based data from the United States.

Methodology

We used data from patients ≥18 years old who contributed to the National Ambulatory Medical Care Survey between 2006 and 2015. The outcome was CTS identified by the International Classification of Diseases-9-Clinical Modification codes (354.0 and 354.1), and the main independent variable was physician-reported diabetes status. Multivariable logistic regression was used to adjust for confounding variables. Odds ratios (ORs) and 95% confidence intervals (CIs) were reported. Stata v15 was used for all analyses.

Results

Among the patients included in this study (n = 322,092), 13.5% were reported to have diabetes while 0.55% reported CTS. The unadjusted odds of having CTS among patients with diabetes was 0.92 (95% CI: 0.74-1.14; p = 0.447). After adjusting for confounding variables, the association remained not statistically significant (adjusted odds ratio [aOR]: 0.84; 95% CI: 0.65-1.09; p = 0.203). Other variables independently associated with CTS included age 50-59 (aOR: 1.91; 95% CI: 1.49-2.45; p < 0.001), female gender (aOR: 1.31; 95% CI: 1.09-1.58; p < 0.004), and current tobacco users (aOR: 1.32; 95% CI: 1.07-1.63; p < 0.01).

Conclusions

No association was found between DM and CTS in adult ambulatory patients in the United States, but results should be considered in light of potential outcome misclassification.

## Introduction

Carpal tunnel syndrome (CTS) is the most common compression neuropathy in the upper limb [1]. In the general population, the prevalence of CTS is approximately 2.1% for men and 3.0% for women [2]. CTS is characterized clinically by numbness, tingling, and pain in the median nerve distribution [3]. While the exact etiology of CTS is not fully understood, researchers agree that the condition is caused by a compression of the median nerve as it passes through the carpal tunnel of the wrist, leading to ischemia and subsequent segmental demyelination [4].

Diabetes mellitus (DM) has been proposed as a risk factor for CTS. DM is one of the leading causes of disability (affecting 9% of the global population and 10.5% of the US adult population) [1], and is a rapidly growing global health issue affecting 420 million people worldwide. Although the pathogenesis linking DM to CTS is not well understood, studies propose that the cause is multifactorial [4,5]. In the hyperglycemic state, excess metabolism of glucose leads to intracellular sorbitol accumulation in the neuron and the adjacent Schwann cells. Ultimately, this leads to axonal degeneration and segmental demyelination of the nerve, making it more vulnerable to compression with a lower threshold to develop CTS [4].

Studies reporting on the association between DM and CTS vary greatly on the magnitude based on the geographic location where the study took place; the association was reported to be lower in population-based studies than in hospital-based studies [4,6-8]. A study from a university hospital in Turkey found that the odds of CTS was 60 times higher in the DM group compared to the control group (odds ratio [OR]: 60; 95% confidence interval [CI]: 13-246) [6]. Population-based studies in Sweden and Taiwan reported a moderate association between diabetes and CTS [4,7,8] (hazard ratio [HR]: 2.10; 95% CI: 1.65-2.70 for the Swedish study [4]; HR: 1.31; 95% CI: 1.28-1.34 for the Taiwan study [8]). Furthermore, different approaches to address confounders may have contributed to variations in the associations assessed, as several studies assessing type 2 DM and CTS did not find associations once age, gender, and body mass index (BMI) were adjusted for [3,9].

Whether the source of heterogeneity among research findings was due to differences in the target population or unmeasured confounding variables is yet to be assessed. In the present study, we aimed to assess if diabetes status is associated with CTS diagnosis using data from a national sample of adults under ambulatory care in the United States to improve the generalizability of results for community-dwelling US adults.

## Materials and methods

We conducted a secondary analysis of data from the National Ambulatory Medical Care Survey (NAMCS). The NAMCS is a sample of visits to non-federally employed office-based physicians, community health centers, and advanced practice providers (nurse practitioners, physician assistants, and certified nurse midwives) who were primarily engaged in direct patient care. Data were collected by assigning each physician randomly to a one-week reporting period. During this period, data from a systematic random sample of visits were recorded using a computerized patient record form [10].

The study sample consisted of all adults >18 years old who participated in the NAMCS from 2006 to 2015. We chose to study the adult population because pediatric CTS is rare and has a unique etiology [11]. We excluded patients with cervical radiculopathy, brachial plexopathy, and peripheral nerve trauma as these conditions can cause peripheral neuropathy and potentially mask the symptoms of CTS. These conditions were identified based on the International Classification of Diseases-9 (ICD-9) code recorded as other diagnoses related to the visit, including chronic conditions. Lastly, patients with missing or incomplete age, gender, smoking status, and BMI data were excluded from analyses.

The primary independent variable was current diabetes status on record, collected systematically by the NAMCS. The dependent variable was patient-reported diagnosis of CTS listed as a chronic condition or diagnosis related to the visit (noted as ICD-9-Clinical Modification code 354.0). Other variables known to influence the risk of carpal tunnel such as age, sex, BMI, obesity, smoking status, race, ethnicity, and history of hypothyroidism, chronic kidney disease, rheumatoid arthritis, psoriatic arthropathy, and asthma were assessed for their potential role as confounders.

Descriptive analysis was performed to assess the characteristics of the sample, followed by bivariate analysis to assess the distribution of the selected characteristics according to diabetes and CTS status. Multivariable logistic regression models were finally used to determine the association between diabetes and CTS. Results were presented as OR and the corresponding 95% CI. P-values less than 0.05 were considered statistically significant. Statistical analyses were performed using Stata version 15 software (StataCorp LLC, College Station, Texas, USA).

This study was based on a secondary analysis of data obtained from the NAMCS database, a publicly available, de-identified dataset. Approval by an internal review board (IRB) and informed consent were not required as the study constituted non-human subject research.

## Results

Approximately 403,703 adults >18 years old participated in the NAMCS from 2006 to 2015 and were eligible for inclusion in the study. Of those individuals, 81,611 were excluded due to missing or incomplete age, gender, smoking status, and BMI data. In the remaining sample, an additional 865 patients with a diagnosis of cervical radiculopathy, brachial plexopathy, and peripheral nerve trauma were excluded. The final sample size consisted of 322,092 individuals. Of these individuals, the prevalence of diabetes was 13.5% (Table 1). Patients who had diabetes were older, more frequently males, non-white, non-current tobacco users, obese, and with chronic kidney disease, hypertension, and asthma. Of note, approximately 17.7% of the group with diabetes were obese, while only 6.7% of the group without diabetes were obese.

**Table 1:** Baseline characteristics of patients with diabetes versus without diabetes. CKD, chronic kidney disease “Other” is defined as Asian, Native Hawaiian, or other Pacific Islander, American Indian, or Alaska native “Not current” is defined as never, former, or unknown prior tobacco use

	Diabetes		No diabetes		P-Value
Characteristics	Number	%	Number	%	
Age					<0.001
18-29	832	1.86	35,147	13.1	
30-39	1,891	4.26	35,997	13.3	
40-49	4,284	9.77	43,538	15.9	
50-59	8,840	20.3	52,120	18.6	
60+	27,674	63.8	111,769	39.1	
Sex					<0.001
Female	23,018	53.1	168,379	62.1	
Male	20,503	46.9	110,192	37.9	
Race					<0.001
White	35,154	79.7	239,856	84.8	
Black	5,786	14.2	25,969	9.96	
Other	2,581	6.15	12,746	5.2	
Tobacco					<0.001
Not current	26,777	84.3	156,964	82.8	
Current	5,711	15.7	34,692	17.2	
Obesity					<0.001
No	36,072	82.3	261,042	93.3	
Yes	17,529	17.7	7,449	6.72	
Hypothyroidism					0.007
No	42,749	97.9	274,727	98.3	
Yes	772	2.09	3,844	1.74	
CKD					<0.001
No	43,066	99	277,818	99.7	
Yes	455	1.01	753	0.28	
Rheumatoid arthritis					0.273
No	43,375	99.5	277,370	99.4	
Yes	146	0.46	1,201	0.56	
Psoriatic arthritis					0.261
No	43,506	100	278,442	99.9	
Yes	15	0.04	129	0.06	
Hypertension					<0.001
No	15,093	34	206,258	72.6	
Yes	28,428	66	72,313	27.4	
Asthma					<0.001
No	40,344	93.1	262,882	94.4	
Yes	3,177	6.91	15,689	5.61	

The prevalence of CTS was 0.55%. As age increased, there was an increasing trend in the frequency of CTS as 0.18% of young adults aged 18-29 had CTS compared to 0.80% of older adults aged 50-59. The proportion of patients with CTS was statistically significantly higher among females (0.57% compared to 0.43% in males), tobacco users (0.68% compared to non-smokers 0.50%), and among those not reporting a diagnosis of hypothyroidism or chronic kidney disease (Table 2).

**Table 2:** Characteristics of patients by diagnosed CTS status. CKD = chronic kidney disease; CTS = carpal tunnel status “Other” is defined as Asian, Native Hawaiian, or other Pacific Islander, American Indian, or Alaska native “Not current” is defined as never, former, or unknown prior tobacco use

	CTS		No CTS		P-Value
	Number	%	Number	%	
Diabetes					0.446
No	1,521	0.52	277,050	99.5	
Yes	254	0.48	43,267	99.5	
Age					<0.001
18-29	90	0.18	35,889	99.8	
30-39	212	0.53	37,676	99.5	
40-49	351	0.65	47,471	99.4	
50-59	464	0.80	60,496	99.2	
60+	658	0.43	138,785	99.6	
Sex					<0.001
Female	1,177	0.57	190,220	99.4	
Male	598	0.43	130,097	99.6	
Race					0.693
White	1,533	0.51	273,477	99.5	
Black	193	0.57	31,562	99.4	
Other	49	0.45	15,278	99.6	
Tobacco					0.005
Not current	910	0.50	182,831	99.5	
Current	292	0.68	40,111	99.3	
Obesity					0.54
No	1,655	0.51	295,459	99.5	
Yes	120	0.57	24,858	99.4	
Hypothyroidism					0.002
No	1,768	0.52	315,708	99.5	
Yes	7	0.13	4,609	99.9	
CKD					0.018
No	1,773	0.52	319,111	99.5	
Yes	2	0.10	1,206	99.9	
Rheumatoid arthritis					0.797
No	1,767	0.52	318,978	99.5	
Yes	8	0.58	1,339	99.4	
Psoriatic arthritis					0.114
No	1,772	0.52	320,176	99.5	
Yes	3	1.46	141	98.5	
Hypertension					0.234
No	1,259	0.54	220,092	99.5	
Yes	516	0.48	100,225	99.5	
Asthma					0.824
No	1,684	0.52	301,542	99.5	
Yes	91	0.50	18,775	99.5	

The odds of CTS in both the unadjusted and adjusted models did not differ by diabetes status. Incidentally, patients aged 50-59, females, and current tobacco users had higher odds of CTS (Table 3).

**Table 3:** Unadjusted and adjusted OR for the diagnosis of CTS. CKD = chronic kidney disease; CI = confidence interval; CTS = carpal tunnel syndrome; OR = odds ratio Logistic regression was performed to obtain adjusted ORs for the following variables because of association with either the exposure or the outcome: age, sex, tobacco use, obesity, hypothyroid, CKD “Not current” is defined as never, former, or unknown prior tobacco use

	Unadjusted		Adjusted	
	OR (95% CI)	P-Value	OR (95% CI)	P-Value
Diabetes				
Yes	0.92 (0.74-1.14)	0.447	0.84 (0.65-1.095)	0.203
No	Reference		Reference	
Age				
18-29	0.42 (0.30-0.59)	<0.001	0.39 (0.27-0.56)	<0.001
30-39	1.22 (0.89-1.67)	0.21	1.1 (0.73-1.66)	0.637
40-49	1.51 (1.18-1.93)	0.001	1.29 (0.94-1.77)	0.116
50-59	1.86 (1.52-2.27)	<0.001	1.91 (1.49-2.45)	<0.001
60+	Reference		Reference	
Sex				
Female	1.31 (1.13-1.53)	0.001	1.31 (1.09-1.58)	0.004
Male	Reference		Reference	
Tobacco				
Not current	Reference		Reference	
Current	1.36 (1.09-1.69)	0.006	1.32 (1.07-1.63)	0.01
Obesity				
Yes	1.12 (0.78-1.60)	0.54	1.18 (0.78-1.79)	0.442
No	Reference		Reference	
Hypothyroid				
Yes	0.25 (0.09-0.66)	0.005	0.21 (0.065-0.71)	0.011
No	Reference		Reference	
CKD				
Yes	0.20 (0.05-0.88)	0.034	0.19 (0.027-1.40)	0.103
No	Reference		Reference	

## Discussion

We found no difference in the odds of DM and CTS diagnoses. This result contributes to the growing body of literature that suggests no association between DM and CTS [3,9]. Examples include a retrospective case-control study of patients from a single institution in the Netherlands where type 2 DM was not a significant predictor for CTS (OR: 0.99; 95% CI: 0.66-1.47) after adjusting for age, gender, and BMI [3]. Similarly, in patients from a single institution in the United Kingdom, the association between DM and neurophysiological abnormalities was not significant after adjusting for age, sex, and ethnic origin (OR: 1.6; 95% CI: 0.9-3.1) [9]. Our findings build from previous studies as it used US nationwide data that allow for better generalizability to adults over 18 years old in the United States.

The pathogenesis of CTS remains complex, and the impact of diabetes on CTS is still unclear and multifactorial. It is thought that diabetes may aggravate ischemia in nerves via glycosylation end products that are under chronic stress of endoneurial hypoxia [4,5]. Other proposed mechanisms include increased extracellular fluid, demyelination of Schwann cells, modification of fibroblasts, and proliferation of cytokines [4]. In the literature, there exist contradicting results regarding DM as a risk factor for CTS. Previous studies that investigated the relationship suggest that the magnitude of the association is fairly modest [1]. However, several studies have shown that there was no association between glycemic control and CTS, with one showing an inverse relationship [12,13]. Gamstedt et al. found that poor metabolic control yielded less risk than the control group, but the finding was not statistically significant (OR: 0.34; 95% CI: 0.10-1.12; p = 0.08) [13]. This was likely due to the period of time that was used to classify metabolic control. Therefore, our results do not support the use of diabetes as a clinical predictor of CTS.

The lack of association reported might be because we did not assess whether diabetes was well controlled or uncontrolled or the effect of medication usage. It is possible that the association between diabetes and CTS only exists in specific subgroups (e.g., uncontrolled diabetes). Additionally, our study cannot exclude the possibility of underreporting of CTS, as evidenced by the low prevalence of the condition compared to previous studies [3,4]. Furthermore, the database only collected ICD-9 information on a maximum of three chronic conditions or diagnoses related to the visit per patient. Thus, a diagnosis of CTS may have been excluded if a patient reported other medical conditions or was inaccurately coded. Lastly, the NAMCS is a national probability sample of physicians that reports on a sample of outpatient visit encounters and not of patients. Therefore, the database estimates the prevalence of conditions per visit and not per patient. Thus, patients requiring frequent visits to the same physician could have contributed to data on more than one visit.

Previous studies that reported the magnitude of effect between DM and CTS varied greatly based on the geographic location of the study, with a lower effect in population-based studies compared to hospital-based studies. Our study encountered a similar finding, with far fewer visits for CTS compared to diabetes. This can partially be explained by sampling bias because patients who experience symptoms of CTS may not seek ambulatory care. Alternatively, it is possible that surgeons took a more conservative approach with non-diabetic patients who had CTS, whereas diabetic patients with CTS were more likely to get carpal tunnel release surgery with less need for maintenance follow-up appointments. This could lead to the differences in outpatient visits between the two groups, which could partially explain the findings in our analysis.

To our knowledge, this study is the first to investigate the relation between CTS and diabetes in a population-based setting in the United States. Our study utilized the NAMCS database, which encompasses 84% of all ambulatory visits in the United States. Compared with direct observation of outpatient visits, the physician reporting method used in the NAMCS was found to be more accurate for procedures and examinations [14].

## Conclusions

Our study found no association between DM and CTS in adult ambulatory patients in the United States. However, the limitations we addressed may have impacted our findings. Future population-based studies utilizing a prospective study design and a larger sample size should be conducted to investigate the association between hemoglobin A1c and CTS to assess whether well-controlled or uncontrolled diabetes plays a role. Hemoglobin A1c level is generally accepted as a more accurate measure of diabetes control as well as an individual’s health risks associated with diabetes. Furthermore, combining additional NAMCS, emergency, and outpatient department data from multiple years would increase the sample size and produce a more comprehensive sample of ambulatory care visits.
